# Tumor-on-a-chip: from bioinspired design to biomedical application

**DOI:** 10.1038/s41378-021-00277-8

**Published:** 2021-06-21

**Authors:** Xingxing Liu, Jiaru Fang, Shuang Huang, Xiaoxue Wu, Xi Xie, Ji Wang, Fanmao Liu, Meng Zhang, Zhenwei Peng, Ning Hu

**Affiliations:** 1 The First Affiliated Hospital of Sun Yat-Sen University, State Key Laboratory of Optoelectronic Materials and Technologies, Guangdong Province Key Laboratory of Display Material and Technology, School of Electronics and Information Technology, Sun Yat-Sen University, 510006 Guangzhou, China; 2 State Key Laboratory of Transducer Technology, Chinese Academy of Sciences, 200050 Shanghai, China

**Keywords:** Electronic devices, Microfluidics

## Abstract

Cancer is one of the leading causes of human death, despite enormous efforts to explore cancer biology and develop anticancer therapies. The main challenges in cancer research are establishing an efficient tumor microenvironment in vitro and exploring efficient means for screening anticancer drugs to reveal the nature of cancer and develop treatments. The tumor microenvironment possesses human-specific biophysical and biochemical factors that are difficult to recapitulate in conventional in vitro planar cell models and in vivo animal models. Therefore, model limitations have hindered the translation of basic research findings to clinical applications. In this review, we introduce the recent progress in tumor-on-a-chip devices for cancer biology research, medicine assessment, and biomedical applications in detail. The emerging tumor-on-a-chip platforms integrating 3D cell culture, microfluidic technology, and tissue engineering have successfully mimicked the pivotal structural and functional characteristics of the in vivo tumor microenvironment. The recent advances in tumor-on-a-chip platforms for cancer biology studies and biomedical applications are detailed and analyzed in this review. This review should be valuable for further understanding the mechanisms of the tumor evolution process, screening anticancer drugs, and developing cancer therapies, and it addresses the challenges and potential opportunities in predicting drug screening and cancer treatment.

## Introduction

Cancer continues to be a leading cause of mortality worldwide, with an estimated 18.1 million new cancer cases and 9.6 million cancer-related deaths reported in 2018^1,2^. By 2040, worldwide cancer cases will increase by 60%, while the number will be ~81% in developing countries, according to the latest report of the World Health Organization (WHO)^3^. The high mortality and morbidity associated with cancers are burdens on global health, highlighting the need to discover and develop more effective anticancer therapies. Over the past half century, many promising drug candidates have been identified for the treatment of cancer. However, the success rate of anticancer drugs in clinical trials is very low, with over 80% of drug candidates failing during clinical screening due to weak efficacy or adverse events^4–7^. Moreover, the cost of new drug development has markedly increased, and drug efficacy and toxicity studies are still costly^8,9^. Moreover, the development process of taking new drugs from a target compound to a marketed medicine is inefficient. Even after drugs are approved for clinical treatment, they can be recalled because of unrevealed side effects, such as severe cardiac, liver, or kidney toxicity, which cause serious health threats for many patients^10,11^. One major reason for these issues is that the results of animal experiments cannot directly verify the toxicity and side effects of drugs in humans due to species differences^12,13^. These differences strongly suggest that the current disease and drug screening preclinical models possess certain predictive defects^13^.

Conventional preclinical models for anticancer drug screening are mainly divided into two categories: an in vitro cell model (2D cell monolayers and three-dimensional cell models) and an in vivo animal model. 2D cell monolayers and three-dimensional cell culture models have been widely employed as initial screening models to provide a cost-efficient and simplified method to elucidate the mechanism of cancer biology or identify the efficacy and safety of a drug candidate^14–16^; however, they are unable to recapitulate complex biochemical and biophysical factors of the tumor microenvironment (TME) in vivo because they lack the systemic nature of tumors, leading to weak predictive ability^17–24^. Tuncer et al. explored drug inhibitory effects (IC_50_) in monolayer (2D) and spheroid (3D) cultures, indicating that monolayer cell cultures may provide misleading results, since the produced IC_50_ values were almost identical for several cases in which spheroid cultures resulted in significantly distinct IC_50_ average values^25^. The reason is that in these experiments, cells are grown in a monolayer, all well exposed to the drug, while in vivo tumors expand as three-dimensional multicellular masses, where inner cells have limited drug exposure. 3D models are able to better reflect the real tumor setting with a more natural response to different soluble factors present in the TME. There are a variety of existing 3D methods, so it is important to identify the most appropriate one to study particular cellular and physicochemical aspects of the TME. Alternatively, animal models, as the gold standard in cancer biology, can imitate the TME to provide essential information regarding tumor growth and tumor response to drug compounds in vivo for the study of the pharmacokinetics and mechanism of action after drug treatment^26–30^. However, in vivo animal models still possess limitations. Species-specific differences between animals and humans cause differences in drug efficacy and toxicity tests in physiology and cell biology^31–37^. In addition, animal studies are hindered by animal expenses, low‐throughput drug optimization and ethical controversy^31–35,38^. Seymour and Ghert et al. demonstrated that obtaining concordance between animal models and clinical trials remains challenging, with an average rate of concordant results that barely reaches 8%^21,39,40^. Thus, a more reliable and predictable screening platform is urgently needed to precisely recapitulate the human tumor model and thereby further understand the complex nature of cancer and the effects of anticancer therapeutics in humans in order to develop effective therapeutic anticancer agents.

Recently, organ-on-a-chip techniques have emerged to recapitulate the microphysiological function and three-dimensional microstructure of in vivo human organs^13,14,41–49^. Organ-on-a-chip devices are microfabricated cell cultures that combine the advantages of microfluidic technology and 3D cell culture technology to mimic the complexity and characteristics of native organs^49–54^. Organ-on-a-chip devices possess good performance and improved recapitulation of the native microenvironment, and they allow high‐throughput tests with decreased cost, ethical advantages and enhanced reproducibility^47,55–57^. They were initially fabricated using micromanufacturing tools such as photolithographic etching adapted from computer microchip fabrication to control the shapes and sizes of surface features at the nano-or microscale to enable sensing and response from living cells in their natural tissue environment^49,58^. Microfluidic culture systems are usually manufactured by “soft lithography”, which is a method of replicating the patterns etched into a silicon template in more biocompatible and flexible materials by casting^41,49,59^. Through precise microfluidic designs, microfluidic devices can reconstruct physiological dynamic characteristics in tissues, such as physiological flow, shear stress, nutrient delivery, and drug action, in a controllable environment, providing a platform for cell culture, microenvironment creation, and organ simulation or in vitro assessment of organ tissue^60–67^. Furthermore, microfluidic systems can link different organs-on-a-chip to potentially simulate the complexity of multiorgan drug metabolism and pharmacokinetics^9,47,50,68–71^. Currently, many types of healthy and diseased tissues and organs, including microvascular obstructions, cystic fibrosis models, heart^72,73^, kidney^74–76^, liver^77–79^, lung^79,80^, pancreas, brain^81–86^, skin^87–89^, eye^90,91^, intestine^92^, and neuropsychiatric disorder models^93–95^, have been constructed on microfluidic chips. Crucially, these organ chip microdevices are able to recapitulate organ-level responses to toxins^79,96,97^, drugs^86,98^, radiation^99^, cigarette smoke^100^, pathogens^96^, and normal microorganisms^96^, as well as the dependence on flow-circulating immune cells and organs to recruit specific in vitro inflammatory responses^101^, which are very important for preclinical drug screening and disease modeling.

Based on the advances of organ-on-a-chip platforms and dynamic culture systems, organ-on-a-chip technology has also been applied to construct 3D models of human tumors in vitro, providing new opportunities for oncology research^14,102–105^. Tumor-on-a-chip has become an attractive prospect in organ-on-a-chip research for studying both cancer biology and treatment options^13,106–108^. It makes use of microfluidics and cell culture technology in a bioinspired design to mimic the interactions between in vivo human tumors and tissues and organs. Tumors possess a complicated microenvironment with a dense extracellular matrix (ECM), irregular vessels, assorted stromal, immune and inflammatory cells, cancer stem cells, and limited perfusion of nutrients^109–112^. Various studies have demonstrated that complex components can significantly affect the growth and metastasis of cancer via mechanical and biochemical factors^113–116^. Therefore, to further investigate cancer development, we should not only study cancer cells in isolation but also study the interactions with associated cells and ECM changes in the TME. Ideally, tumor-on-a-chip platforms have the ability to recreate many cardinal TME features and show great promise as a novel technology for studying the mechanisms of tumor evolution, screening anticancer drugs and cancer therapies, and enabling precision medicine^14,112,117–122^.

**Fig. 1:**
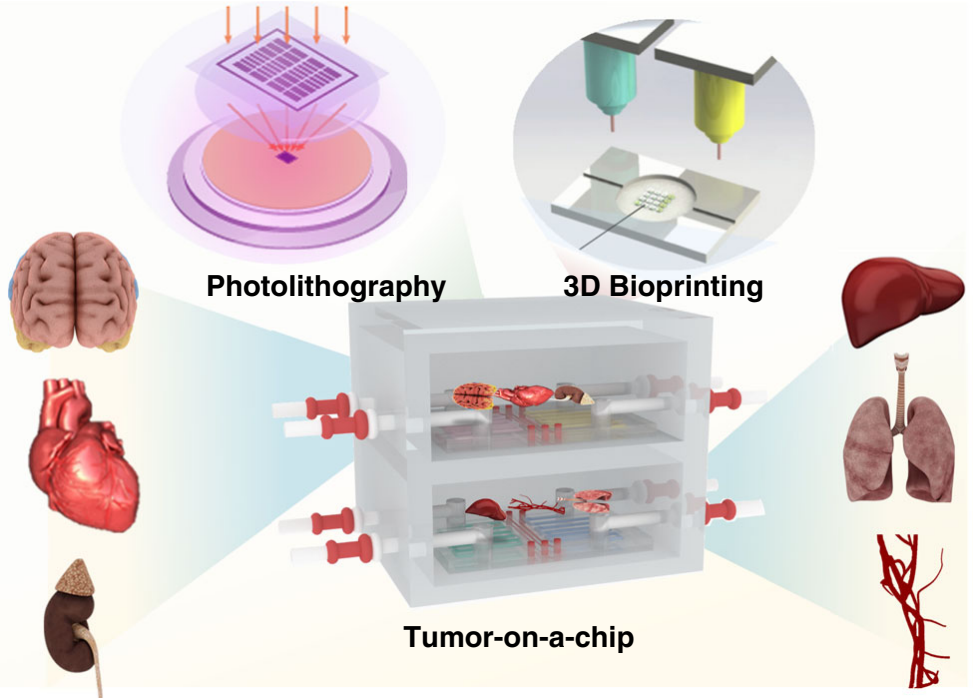
Schematic of the tumor-on-a-chip platform for modeling tumor cell tissue structure and functional units in vitro. Tumor-on-a-chip platforms can be generally prepared by photolithography and 3D bioprinting and applied in cancer biology and anticancer therapy research.

Some previous reviews have already focused on tumor-on-a-chip devices from a variety of perspectives. Hachey and Hughes investigated the pros and cons of standard preclinical models, the tumor microenvironment, microphysiological systems for cancer research and the applications of tumor chips^123^. Kumar and Varghese reviewed ex vivo tumor-on-a-chip platforms to study intercellular interactions within the tumor microenvironment^124^. Wang et al. devised tumor-on-a-chip platforms for assessing nanoparticle-based cancer therapy^108^. LeDuc et al. reviewed some recent achievements in tumor-on-a-chip approaches and presented potential future directions for tumor-on-a-chip systems in areas including mechanical and chemical mimetic systems^125^. These reviews mainly focused on the applications of tumor-on-a-chip platforms, such as drug screening for cancer therapies and tumor mechanisms. However, the technology to fabricate tumor-on-a-chip platforms is rarely discussed. In this review, we will not only introduce the latest developments of tumor-on-a-chip microfluidic devices in cancer biology studies and biomedical applications with microfluidics but also present microfabrication, biomaterials, and tissue engineering technologies to recapitulate TMES (Fig. 1). Strategies are first discussed, such as the fabrication technology and materials used in the tumor-on-a-chip model. Subsequently, the different tumor-on-a-chip types used to reconstitute human organs/tissues in vitro are presented, including lung tumor chips, breast tumor chips, brain tumor chips, melanoma tumor chips and tumor metastasis chips. These tumor chips can simulate the 3D microstructure and microphysiological functions of in vivo human organs/tissues, providing a better understanding of the mechanisms of tumor growth and metastasis. Then, we discuss the applications of tumor-on-a-chip systems. Multiorgan-on-a-chip systems that include the interactions of cell cocultures have shown great potential in screening anticancer drugs and cancer therapies. Finally, challenges and potential opportunities of tumor chips are also reviewed. This review will provide a further understanding of the current tumor-on-a-chip developments based on organ-on-a-chip techniques and their applications in cancer biology and anticancer therapies.

## Manufacture of tumor-on-a-chip

To create biomimetic 3D tumor models that better encapsulate pathologic processes, several important factors in the tumor microenvironment must be considered, including oxygen tension, which is a key regulator of cellular behavior. In contrast to the tumor growth microenvironment in vivo, tumor models in vitro are usually under atmospheric oxygen conditions, which are not representative of the conditions in vivo. Therefore, the results of these studies can be misleading. In an in vitro metastasis model, migrated tumor cells must be further exposed to varying microenvironments and oxygen gradients to simulate the process of in vivo intravasation^126^. Chips based on microfluidics can be designed to realize oxygen gradients with high spatial and temporal resolution, which can be used to simulate the physiological effects of oxygen on tumor progression and metastasis^127,128^.

### Principle and fabrication of tumor-on-a-chip

**Fig. 2:**
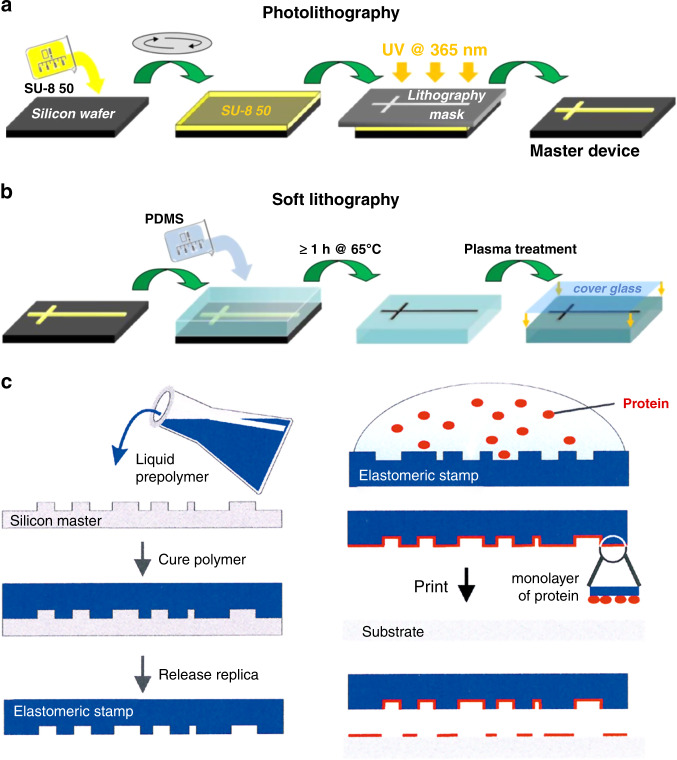
Microfabrication techniques for tumor-on-a-chip. **a**, **b** Use of photolithography and soft lithography to fabricate a simple PDMS microfluidic culture device, conformally sealing it to a flat glass substrate. Adapted with permission^136^. Copyright 2013, RSC. **c** Use of microcontact printing to generate a protein pattern for cell culture. Adapted with permission^144^. Copyright 2000, Wiley.

Tumor-on-a-chip is a miniature cell culture device that simulates tumor cell tissue structure and functional units in vitro. Tumor-on-a-chip can be used to simulate tumor growth and expansion, angiogenesis, and progression from early to advanced lesions involving epithelial–mesenchymal transition (EMT), tumor cell invasion and metastasis^129^. Organ-on-a-chip systems are based on the use of microfluidics to construct tissue models. By using cancer-derived cells and related ECMs in tissue-specific structures to replace those of healthy cells, so-called tumor-on-a-chip systems have emerged. Therefore, the main processing technology of tumor-on-a-chip systems is the same as that of organ-on-a-chip systems. There are several key elements in fabricating an integrated organ-on-a-chip, including a microfluidic system, 2D/3D microtissue culturing, stimulus-loading components, and sensors for monitoring and readout of the results^49,130^. The same is true of tumor chips. Like an organ-on-a-chip, the first step in the construction of a tumor-on-a-chip is to understand the basic elements necessary for the physiological function of the target organ and then determine key features such as different cell types, structures, and the organ-specific physiochemical microenvironment. The second step is to design a cell culture device based on the known characteristics^49^. A variety of techniques have been adopted to fabricate tumor-on-a-chip devices, among which the most widely employed are photolithography, replica molding, soft lithography, microcontact printing and bioprinting technology (Fig. 2)^122,131–136^. Photolithography is a micromanufacturing technology combining with photoresist, mask, ultraviolet light and etching technology^137^. First, masks are required according to the target structures. Then, a layer of photoresist is spin-coated on a substrate that can be corroded by chemical reagents, such as silicon, glass, or quartz, and the photoresist is exposed to UV light. After this step, the pattern is transferred to the substrate, which is finally etched to obtain a microfluid chip with micro flow channels (Fig. 2a)^136^. For tumor-on-a-chip microfluid devices prepared by soft lithography technology, the first step is to prepare a microchannel mold on a silicon substrate by photolithography; the second step is to use a liquid polymer such as PDMS to pour the mold to obtain an optically transparent rubber-like stamp with microstructures; finally, various complex 3D microchannels are obtained on different polymer substrates by transferring the pattern from the stamp (Fig. 2b)^136,138–140^. It is worth mentioning that soft lithography fabrication technology has the ability to change the chemistry of the substrate surface, making it a promising low-cost method for manufacturing microfluidic chips. Replica molding is a subtechnology of soft lithography^49,141,142^. A patterned silicon mold is obtained by photolithography, and then the liquid polymer, such as PDMS, is poured onto it for thermal curing. Subsequently, the PDMS device is peeled off the substrate and sealed and assembled on a flat, smooth substrate such as glass to obtain a microfluidic chip with microfluidic channels. The manufacturing process of microcontact printing is similar to that of replica molding. The only difference is that the PDMS device is further used to control the pattern of cultured cells by printing the PDMS stamp on the substrate with biofunctional molecules such as proteins in a designed pattern (Fig. 2c)^143^. Therefore, the cells on the membrane can also be patterned by controlling the pattern of the printed proteins^144^. Although many tumor chips have been successfully manufactured by photolithography or related manufacturing processes involving photolithography, they still have some shortcomings, such as the need for multiple masks and a multistep photolithography process, which makes the fabrication process time-consuming and costly. Moreover, these methods are capable of fabricating only the microfluidic chip itself, while the other elements (i.e., microtissues, stimulus-loading components, and results-readout sensors) require additional processes^130^.

**Fig. 3:**
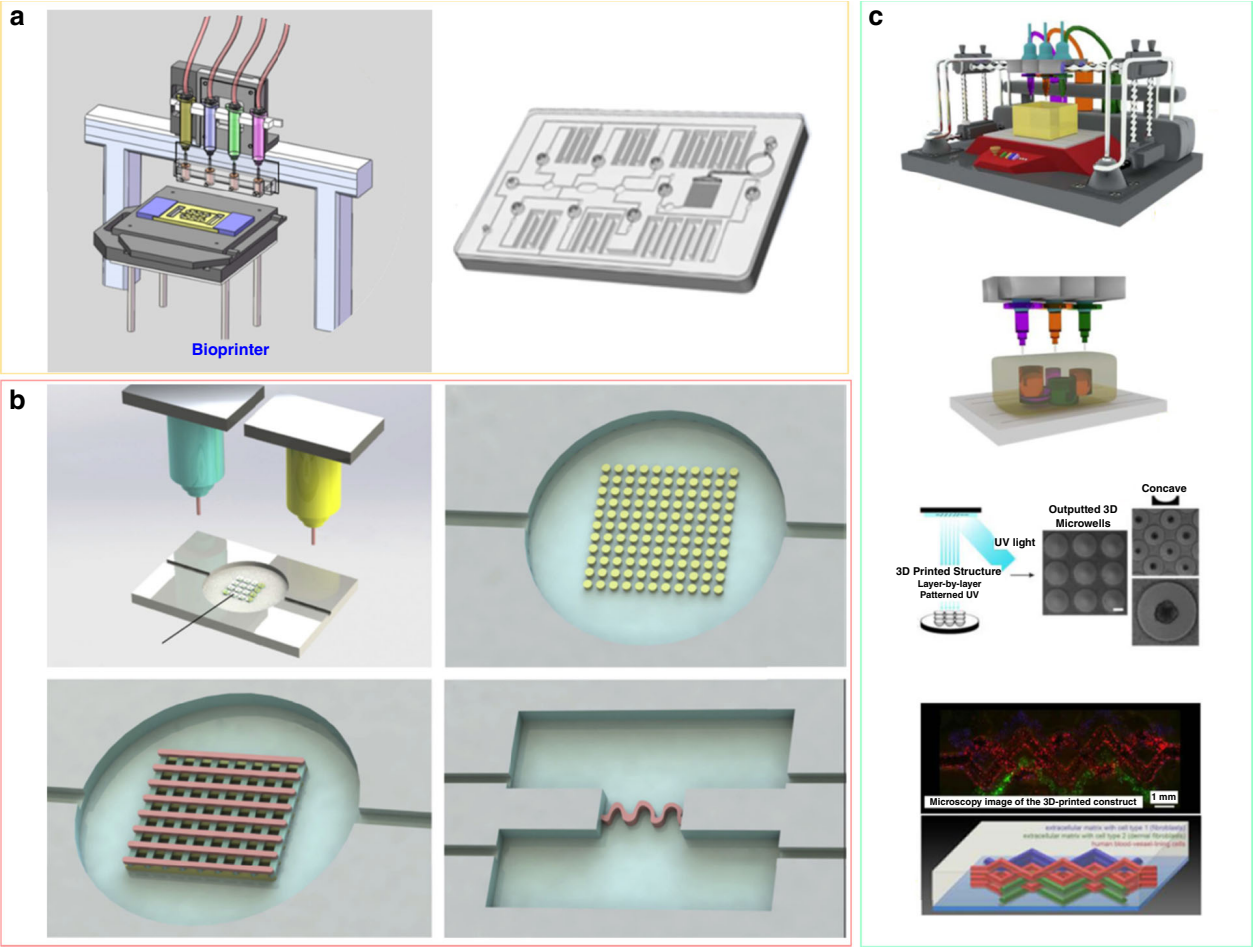
Bioprinting technology for tumor-on-a-chip fabrication. **a** Using 3D bioprinting to create microfluidic chips for 2D/3D microtissue culture. Adapted with permission^130^. Copyright 2017, American Institute of Physics. **b** 3D bioprinting was used to produce different patterns and complex 3D microstructures to model the TME. Adapted with permission^158^. Copyright 2019, Elsevier. **c** Using 3D bioprinting to fabricate heterogeneous tissues: 3D microwells were constructed to facilitate spheroid formation and vascularized tissue models. Adapted with permission^159^. Copyright 2016, Whioce Publishing Pte. Ltd.

In recent years, bioprinting technology based on layer-by-layer printing has been used for tumor-on-a-chip fabrication (Fig. 3)^132,145–147^. Various biofunctional materials and cell types can thus be printed simultaneously onto a substrate of cell-compatible biomaterials to build 3D complex constructs with good spatial resolution and reproducibility (Fig. 3a)^130,148–151^. Bioprinting has different modes, such as fused deposition modeling (FDM), stereolithography (SLA) bioprinting, inkjet bioprinting, and laser-assisted bioprinting. The bioprinting technology used to fabricate tumor-on-a-chip possesses some advantages. First, it has the ability to mimic the heterogeneous microenvironment and complex 3D microstructures of the tumor. It has been a major challenge to realize ECM-like heterogeneous composition in microfluidic channels of tumor-on-a-chip^152^. Based on bioprinting technology, bioinks can create cell aggregates comprising different types of cells, including cancer-associated fibroblasts, immune cells and endothelial cells, to form vascular networks^153–155^. In addition, bioprinting technology is able to create a biomimetic microenvironment for the heterogeneous distribution of biologically relevant proteins and growth factors, which are important to control tumor cell signaling, proliferation, and migration^156,157^. Second, bioprinting technology has the ability to directly print/pattern cells in microfluidic devices, modeling vasculature and biological barriers (Fig. 3b, c)^158,159^. Vascularization is very important to maintain tissue activities and can be used to separate different tissue compartments. The vasculature of tumors is very different from the blood vessels that supply healthy tissues, especially in terms of heterogeneity, permeability, multidirectional blood flow, and unordered distribution throughout the tumor^160^. Successful vascularization has always been a major challenge in the production of functional tissues for tumor in vitro models^161^. A bioprinted blood vessel network can imitate these abnormalities, which could be further used to test and compare the behavior of healthy and abnormal blood vessels under different conditions and treatments^162^. Hence, bioprinting technology has the potential to build miniaturized, multiorgan bionic pathophysiological models, accelerate the pace of research, and promote the application of technology in the medical field.

### Materials for tumor-on-a-chip

Microfluidic tumors-on-chip have been used in drug research and poison testing. The most widely used material for fabricating tumor-on-a-chip devices is polydimethylsiloxane (PDMS). The advantages of PDMS include optical transparency, breathability, biocompatibility, and flexibility, enabling the continuous microscopic observation of tissue constructs for real-time evaluation of cell behavior and response to treatment. In contrast to glass or plastic substrates, devices based on PDMS provide cells with a mechanical environment that is closer to the physical characteristics of soft tissues, possessing porosity and lower rigidity. Despite this, PDMS has a disadvantage, namely, its hydrophobicity, which limits the application of PDMS in chemical screening because PDMS may bind or absorb hydrophobic molecules^163^. To solve this problem, Kang’s group developed a protocol using poly(methyl methacrylate) (PMMA) substrates chemically and robustly bound to porous orbitally etched polyethylene terephthalate (PETE) membranes to construct a microfluidic device that is impermeable to small lipophilic molecules^164^. The results of drug testing demonstrated that human lung adenocarcinoma cells cultured in the PMMA microfluidic device give more reliable results regarding the cytotoxicity of vincristine than human lung adenocarcinoma cells cultured in the polydimethylsiloxane (PDMS) device. This is because the small molecules cannot permeate the PMMA material. This strategy is promising for application to the fabrication of tumor-on-a-chip devices using many different thermoplastic materials and porous track-etched membranes, as it makes it possible to create three-dimensional microstructures that more accurately simulate physiological conditions at the organ level.

In addition to common PDMS and PMMA, there are other materials that can be used to construct microfluidic tumors-on-a-chip. Gelatin is a collagen-derived natural biopolymer. Due to its cellular response characteristics and the ability to deliver a wide range of biomolecules, it is widely used in drug transport models and tissue engineering^165^. Gelatin cannot only promote cell growth but also polymerize with proteins, growth factor nucleotides, polysaccharides and other polyionic complexes. This material has broad prospects for drug transport models due to the controlled sustained and/or targeted release of bioactive molecules. In addition, photocrosslinked gelatin methacrylate (GelMA) hydrogels have been used in corneal tissue engineering, peripheral nerve regeneration and cartilage structure preparation^166^.

Another novel material is bacterial cellulose paper. Bacterial cellulose has the comprehensive advantages of cellulose at the nanoscale and is derived from natural sources, which have good biocompatibility and cost effects. The bacterial cellulose nanofibers are processed and dried to form a stable paper device for building models. Researchers have used this paper equipment to make various microchannels and have successfully established 3D paper vascularized breast tumor models, resulting in the implementation of new materials for simple, low-cost models^167,168^.

Basement membrane extract (BME/Matrigel) is a biomaterial with a composition similar to that of the early conservative basement membrane, which promotes the organization of different tissue types and microtissues of different species. BME/Matrigel, whose main ingredients are laminin-111, collagen IV, entactin, and heparan sulfate proteoglycan, provides structural and signal transduction functions. Under similar conditions, tumor cells have high proliferation and are sometimes aggressive in BME/Matrigel, which can be used to evaluate the malignancy of tumors and explore tumor treatment methods. BME/Matrigel can also enable 3D tumor culture and coculture of other cells, and researchers can adjust the microenvironment to be suitable for tumors by supplementing various factors on BME/Matrigel, which can better inform the mechanism of tumor growth^169^.

### Advanced tumor-on-a-chip

With the development of materials and manufacturing techniques, such as microfluidics and microprinting, combined with stem cell technology, 3D human tissue-like models known as organoids and spheroids are generated^20,170^. They can be used to research human development, disease progression, and treatment, as well as to develop personalized drug methods. Organoids are 3D cell culture models that can self-organize into complex organ-like tissues. Organoid models are usually generated from stem cells, including embryonic stem cells (ESCs), induced pluripotent stem cells (iPSCs), and adult stem cells^171^. Organoids derived from these stem cells can be integrated into tumor chips, and because of the combination of the two technologies, organ-specific structures and gene expression signatures in vivo could be mimicked better than in either model alone^172^.

**Fig. 4:**
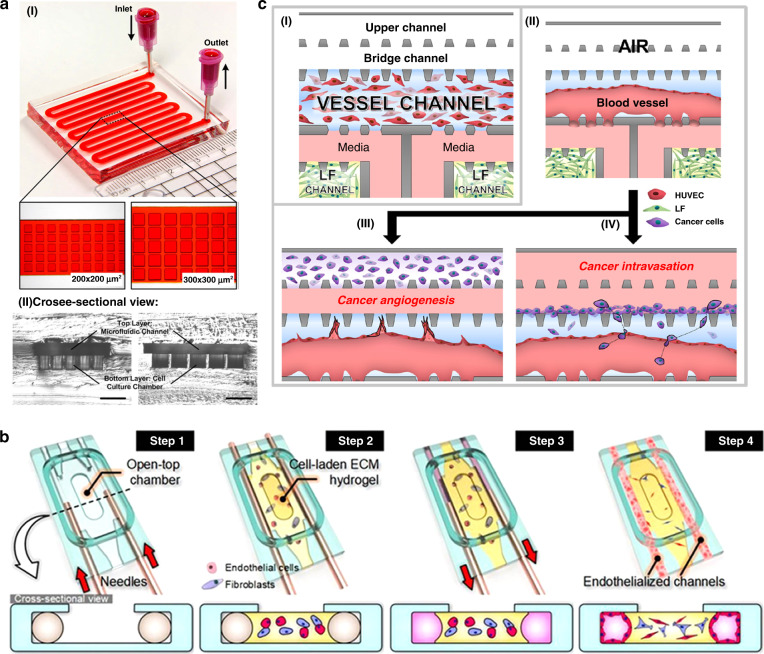
Advanced tumor-on-a-chip models. **a** Microfluidic device for drug testing and flow cytometry analysis of tumor spheroids. I Two-layered microfluidic devices with different culture chamber geometries to prepare spheroids of different sizes for drug testing. II Microscopic cross-sectional views of microfluidic devices with different culture chamber geometries. Scale bar is 500 μm. Adapted with permission^178^. Copyright 2016, Springer Nature. **b** A microfluidic platform of perfusable microvascular beds to produce vascularized three-dimensional human microtissues. Adapted with permission^182^. Copyright 2016, Springer Nature. **c** A microfluidic metastasis chip platform for microvessel formation to analyze cancer angiogenesis and intravasation. I Schematic diagram of the microfluidic metastasis chip. II Microvessel formation. III Cancer angiogenesis. IV Cancer intravasation. Adapted with permission^183^. Copyright 2016, Springer Nature.

A cell spheroid is a three-dimensional (3D) aggregation of multiple single cells, which are obtained from cancer cell lines or dissociated cell clusters from tumor tissue in nonadherent substrates^173^. A 3D coculture model of tumor spheroids has been developed to study different in vitro cancer types, such as lung and salivary gland cancers, as well as invasion and extravasation, as cell migration leads to infiltration and extravasation events during metastasis (Fig. 4)^67,174^. It can be used to mimic in vivo tumor microenvironments because it has the ability to provide metabolism, proliferation, and species concentration gradients similar to those found in vivo^175,176^. Based on spheroids, Sung’s group proposed a 3D microfluidic model of breast cancer invasion using surface tension pumping to achieve the sequential loading of cells at different time points^177^. Employing this device, the researchers proved the relevance of tumor invasion and cell migration assays in breast cancer biology, showing good consistency in tumor invasion studies in in vivo xenograft models. The 3D tumor spheroid is one of the well-characterized approaches of 3D in vitro cell culture models to improve the predictive capacity for preclinical drug testing. Patra et al. used a microfluidic device to perform drug detection and flow cytometry analysis on a large number of uniformly sized tumor spheroids (Fig. 4a)^178^. The experimental results showed the importance of drug detection in three-dimensional tumor spheroid models. In addition, the cell culture format (two-dimensional monolayer versus three-dimensional spheroid) and spheroid size play key roles in drug responses and show the advantages of connecting these two technologies in drug screening applications. These results have great potential to help pharmaceutical researchers better understand the functions of drugs in more in vivo-like 3D cell structures.

The microvascular system plays an important role in the circulatory system, in which blood flows: the microvascular system provides nutrition and removes waste products from the body^179^. Researchers have used organ-on-a-chip technology to construct a microvascular system with selective barrier function similar to that in the body to simulate the microstructure and microphysiological functions of human organs to better describe organ functions and characteristics^179^. The organ-on-a-chip microvascular model is usually constructed by the self-morphogenesis method; that is, endothelial cells are added to the surface of a cylindrical cavity to reconstruct the vascular system in the 3D cavity^180^. In addition, there is a method to produce self-assembled vascular networks. Recently, the introduction of vascular networks with surrounding tissues in microdevices has been reported^181^. A 3D controlled perfusion microvascular network was built by using poly(dimethylsiloxane) (PDMS) and multiple compartments. This device usually consists of an upper medium reservoir and parallel microchannels on both sides. When planting cells, a long needle is first inserted into each microchannel past the inlet access ports oriented sideways, and the ECM hydrogel precursor is injected, followed by the suspension and mixing of endothelial cells and fibroblasts in the cell culture chamber. Enzymatic curing then forms a cellular hydrogel scaffold. After the gel is formed, the needle is removed to form a circular channel for cell implantation (Fig. 4b)^182^. This technology is used to study the construction of heart models, the construction and pathogenesis of atherosclerosis models, the mechanism of thrombosis and the interactions between blood components and vascular endothelial cells^180^. The organ-on-a-chip technology of the microcirculation system has also been applied to tumor research. Tumor blood vessels have a great effect on the occurrence and development of tumors; therefore, the microfluidic technology of organ chips is also used to study the formation of tumor blood vessels. The researchers cocultured tumor cells and ECMs in a microfluidic system and established vertical 2D monolayer endothelial cells on the sidewall of the model to promote the generation of axial planar blood vessels into ECMs. In this model, researchers found that highly malignant human glioblastoma can induce abnormal morphology of blood vessels. Lee et al. also proposed a tumor angiogenesis model to quantify the angiogenesis of new microvessels (Fig. 4c)^183^. The goal of the research on tumor migration is mainly to study the interaction between the tumor and stroma, mostly based on coculture research and a cell model in the form of a separate microfluidic chamber.

Since the tumor-on-a-chip system is usually composed of cells from a single organ, its recombination ability is limited. To solve this problem, researchers developed multiorgan-on-a-chip systems to evaluate the efficacy and off-target toxicity of anticancer therapeutics. These systems combine the mature organ-on-a-chip system with functional organ modules; each module is optimally developed and assembled into a whole when the function matures^102,184^. Du et al. explored the absorption, metabolism and toxicity of ginsenoside compound K utilizing multiorgan chips based on vascular, intestinal, kidney, and liver chips^185^. Sung’s group developed a pumpless multiorgan-on-a-chip platform combined with a mathematical pharmacokinetic–pharmacodynamic model to investigate the mechanism of action of drugs with interactions between multiple organs^186^. By reproducing the dynamics of multiorgan interactions, multiorgan-on-a-chip systems can be used to study various dynamics of diseases and drug activities in mechanistic detail.

## Different types of tumor-on-a-chip

A variety of human-derived in vitro tumor-on-a-chip models have been developed for studying various characteristics associated with tumor progression, such as growth, angiogenesis, metastasis, and drug response. By combining advanced microfabrication techniques, such as photolithography, soft lithography and bioprinting, with microfluidic and tissue engineering techniques, tumor chips can ideally reproduce specific key aspects of the tumor microenvironment, such as biochemical gradients and niche factors, dynamic cell–cell and cell–matrix interactions, and complex tissue structures comprised of tumor and stromal cells. Here, we specifically highlight some cancer models with high morbidity and fatality rates, including lung tumor chip, liver tumor chip, brain tumor chip, colorectal-tumor chip, breast tumor chip and pancreatic tumor chip systems (Table 1).

**Table 1:** Summary of the different types of tumor-on-a-chip

Tumor-on-a-chip models	References	Cell types	Drugs	Applications
Lung tumor chip	^98^	Human NSCLC cell line	Tyrosine kinase inhibitors	Explain the high level of resistance to therapy in lung cancer patients and provide an experimental model to study cancer persister cells and mechanisms of tumor dormancy in vitro
	^131^	Human alveolar epithelial cells and human pulmonary microvascular endothelial cells	Nanoparticles	Reconstitute the critical functional alveolar–capillary interface of the human lung to response to bacteria and inflammatory cytokines introduced into the alveolar space
Brain tumor chip	^196^	Brain tumor stem‐like cells		Examine the function of primary patient‐derived BTSCs
	^197^	Glioblastoma cells (U87)	Pitavastatin Irinotecan	Develop a 3D brain cancer chip for drug screening
	^198^	C6 glioma cells	Colchicines	Study the cellular response to different concentrations of colchicines
Liver tumor chip	^189^	HepG2/C3A MEG‐01 MES‐SA MES‐SA/DX‐5	Doxorubicin Cyclosporine Nicardipine	Provide a combined strategy for selectively inhibiting MES‐SA/DX‐5 cell proliferation; may prove to be advantageous in vivo by specifically targeting MDR cancer with acceptable side‐effects
	^190^	iPS C2a Functional endothelial cells Cardiomyocytes hepatocytes		Refine microtissues, establish modular multitissue platforms, and study interactive responses of cardiac, vascular and hepatic microtissues to pharmacological agents and to physiological and pathological stimuli
Colorectal-tumor chip	^210^	CRC cell line HCT-116	CMCht/PAMAM nanoparticle	Evaluate precision nanomedicine delivery
Breast tumor chip	^211^	MDA-MB-453, MDA-MB-231, and HCC1937	Paclitaxel Olaparib Cisplatin	Develop a microfluidic device for a 3D breast cancer screening platform
Pancreatic tumor chip	^216^	MIA PaCa-2 BxPC3 HT-29		Replicate cellular morphologies and reflect the death of endothelial cells during the metastasis process
	^217^	HepG2s MSCs HUVECs		Provide a suitable platform for pancreatic cancer cell growth for studying the metastasis of pancreatic cancer

### Lung tumor chip

**Fig. 5:**
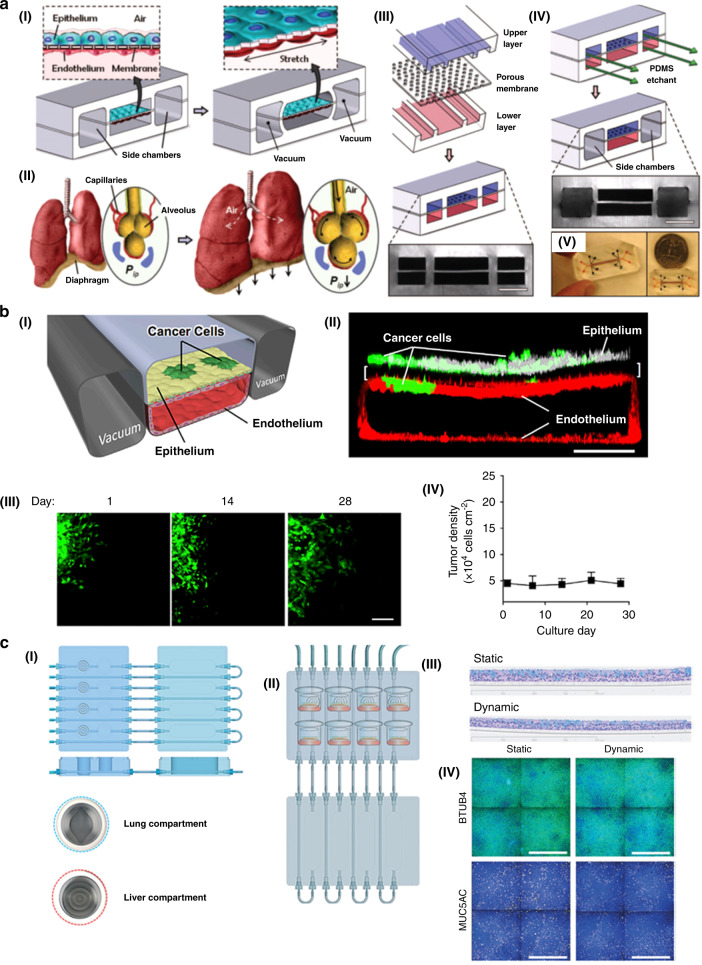
Some examples of lung-on-a-chip models. **a** Biologically inspired design of organ-level lung functions on a chip. I A lung mimic device with an alveolar–capillary barrier was fabricated using PDMS in combination with applying vacuum to the chambers. II Illustration diagram of the interaction of pulmonary alveoli with adjacent capillaries. III Fabrication of a microfluidic device with three parallel microchannels separated by a 10 μm-thick PDMS membrane. Scale bar, 200 μm. IV A PDMS etchant is used to selectively etch the membrane layers to form two large side chambers to which vacuum is applied to cause mechanical stretching. V Photos of an actual lung-on-a-chip microfluidic device viewed from above. Adapted with permission^131^. Copyright 2010, Science. **b** Human orthotopic lung cancer on-a-chip models. I Schematic diagram of a 2-channel microchannel chip coculturing NSCLC tumor cells and human lung microvascular endothelial cells to form a hollow vascular cavity. II Confocal fluorescence micrograph of a cross section of the two central cell-lined channels of an alveolar chip. Scale bar, 200 μm. III Immunofluorescence micrograph of an implanted cluster of GFP-labeled NSCLC cells (green) cultured in an airway chip at 1, 14, and 28 days after implantation. Scale bar, 100 μm. IV Quantification of NSCLC tumor cell densities when cultured for up to 1 month after implantation in the fully differentiated airway chip. Adapted with permission^98^. Copyright 2017, Elsevier. **c** A lung/liver-on-a-chip device for acute and chronic toxicity studies to predict the safety of inhaled compounds. I A schematic diagram of the lung/liver-on-a-chip platform. II The eight pictograms show the location of NHBE ALI tissues placed in the liver chamber and lung chamber for 28 days of culture. The flow control board was connected to the liquid storage board. III Representative histological sections of NHBE ALI tissues after 28 days of culture under static and dynamic conditions. IV Representative images of NHBE ALI tissues maintained under static or dynamic conditions for 28 days stained with β-tubulin 4 (green) and MUC5AC (yellow). Adapted with permission^79^. Copyright 2018, Royal Society of Chemistry.

The lung is a vital organ for gas exchange between external oxygen and carbon dioxide in the blood. It is also one of the most common ports for drugs, toxins, pathogens, and other agents entering the human body. Research on cell–cell, cell-blood flow, and cell-gas flow interactions in the respiratory tract is of great significance for physiological research and drug delivery. Lung cancer is one of the most common cancers and has a high fatality rate. Therefore, it is very important to establish a lung tumor-on-a-chip model to understand the mechanism and treatment of lung cancer (Fig. 5)^79,98^. Typically, the lung-on-a-chip contains two microfluidic channels separated by a porous extracellular matrix in the middle. Human lung epithelial cells are on one side, and pulmonary microvascular endothelial cells are on the other. This model can simulate multiple physiological functions of the lung. The lung tumor chip is based on the lung chip and integrates lung tumor cells into lung epithelial cells. After electroplating, the cell culture fluid is removed from the upper layer of the channel to create a gas-liquid plane, and the nutrient feed is transported through the microvascular channel. Finally, the extent of tumor cell growth and invasion are expressed optically. Through the lung tumor chip, Ingber et al. found that tumor cells were confined to smaller areas during simulated lung periodic breathing movements and that tumor cells spread when the chip stopped simulating breathing movements (Fig. 5a)^131^. Then, Ingber et al. developed a microfluidic chip to fabricate human orthotopic non-small-cell lung cancer models. Through the platform, researchers recapitulated orthotopic lung cancer growth, therapeutic responses, and tumor dormancy in vitro (Fig. 5b)^98^. As a result, researchers believe that tumor cell growth fills the alveolar space and reduces lung breathing movement, which results in positive feedback and promotes tumor cell growth and invasion. Moreover, through lung tumor chips, researchers also found that coculture of tumor cells and alveolar epithelial cells can increase cell–cell interactions, but endothelial cells can inhibit this effect^98^. Hoeng’s group used human bronchial epithelial cells to produce a lung-on-a-chip with a gas-liquid plane and combined it with a hepatocyte-on-a-chip made of liver cells to form a liver/lung-on-a-chip platform (Fig. 5c)^79^. This platform can evaluate cell viability by observing the content of adenosine triphosphate (ATP) and the number of apoptotic necrotic cells. Through this platform, the toxicity of AFB1 to liver–lung crosstalk could be evaluated. AFB1 is a mycotoxin produced by *Aspergillus flavus* and is a common food contaminant in warm and humid countries. Researchers have found that when liver spheroids are present, AFB1 toxicity in the lung-on-a-chip is reduced, demonstrating that liver detoxification can reduce AFB1 toxicity. The liver/lung-on-a-chip platform can evaluate the compound toxicity of drugs and provide new ideas for future drug development.

### Liver tumor chip

The liver, as the largest and main metabolic organ in the body, plays a leading role in several key functions to maintain normal physiological activities, such as controlling blood glucose and ammonia levels, synthesizing various hormones, and detoxifying endogenous and exogenous substances^187^. Its structure is intricate and is mainly composed of two primary cell populations: parenchymal cells and nonparenchymal cells. Parenchymal cells called hepatocytes are functional cells, constituting 80% of all liver cells^188^. They have been the object of investigation of the biological and functional behaviors of liver in vitro systems. Globally, liver cancer accounts for nearly 850,000 new cases each year^187^. The liver tumor chip could provide a platform for liver cancer treatment.

Cytotoxicity assays are very important for drug development and screening. They can predict the direct toxicity of the drug itself or the indirect toxicity to the liver or other organs caused by the liver metabolites of the drug. Adverse reactions caused by drug metabolism increase the role of hepatocytes in organ-on-a-chip systems. There are two major examples of drug metabolism in the liver: the conversion of prodrugs such as thymopeptide component 5 (TF5) to metabolite drugs such as fluorouracil (5-FU), which cause hepatotoxicity and liver injury, and the metabolism of doxorubicin to doxorubicin, which causes hematotoxicity, such as cardiotoxicity and myeloid toxicity^188^. Hence, the liver needs to be cocultured in a microscale cell culture simulation system (μCCA) to study the pharmaceutical aspects of anticancer drugs. Shuler and Tatosian developed a novel liver–intestine–breast platform to mimic the body’s response using interconnected compartments that represent various tissues or organs, which can be used to evaluate drug mixtures for potential efficacy in treating multidrug-resistant cancers^189^. Hirschi’s team exploited integrated heart-liver-vascular microphysiological systems with functionally connected vascular, liver and cardiac microtissues derived from a single line of human pluripotent stem cells for drug testing in human health and disease^190^. Other studies included cocultured liver in their μCCA systems, such as liver–breast–cervical^191^, liver–prostate–kidney^192^, liver–lung–kidney–fat^193^, and liver–lung–fat^194^ systems. Briefly, using μCCA systems as a coculture model with liver cells can be the best candidate to research drug resistance and metastasis of cancers.

### Brain tumor chip

**Fig. 6:**
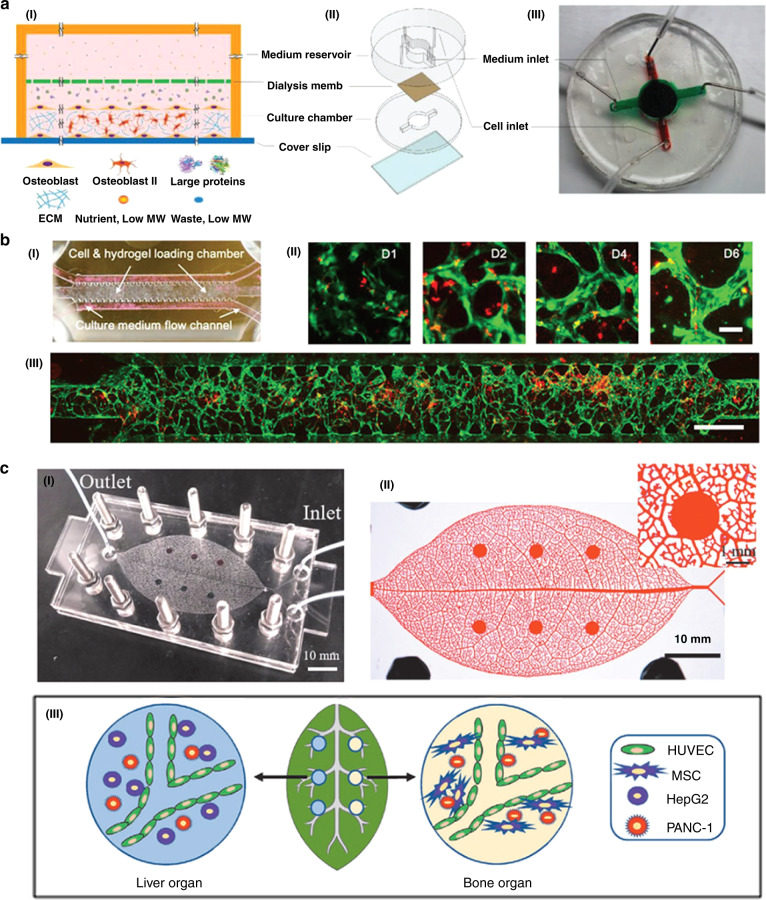
Different types of tumor-on-a-chip platforms. **a** Schematics of the tumor-on-a-chip design for metastatic breast cancer research. The chip is made up of a top culture medium reservoir and a bottom cell growth chamber, separated by a dialysis membrane. Adapted with permission^213^. Copyright 2018, Wiley. **b** Microfluidic device-cultured BTSCs to study glioma. I A schematic diagram of the tumor chip platform, consisting of a cell/gel loading microchamber and two medium flow channels on both sides. II Representative images of microvessel formation at different time points over 6 days. III A whole chip scan showing interconnected microvessel network formation on day 4. Adapted with permission^196^. Copyright 2019, Wiley. **c** Biomimetic vascular microfluidic system on a human-on-leaf-chip for studying organ-specific pancreatic cancer metastasis. I Photograph of the assembled biomimetic vascular human-on-leaf-chip system. II Image of the chambers of interconnected biomimetic vascular networks in the chip filled with red ink. III Schematic diagram of the vascularization of liver and bone tissue in the biomimetic vascular system where PANC-1 cancer cells have metastasized. Adapted with permission^217^. Copyright 2020, Wiley.

The most common and deadly brain tumor is glioma. The transwell-based cell coculture model is a good tool for studying cell movement and interactions. It allows cells to be cultured in a small amount of liquid and is currently used for glioma research^195^. Researchers use the microvascular system on a chip as the perivascular niche (PVN) to study the in vitro kinetics of brain tumor stem-like cells (BTSCs). It was found that the PVN is a niche for BTSCs, and the microvascular diameter can be used as a pathway for tumor metastasis. This model can be used to describe the dynamics and heterogeneity of tumor cells in vivo and represents a new way to study specific tumors (Fig. 6b)^196^. Fan et al. designed a 3D brain tumor chip with PEGDA hydrogel as a carrier for drug delivery and biological applications^197^. They injected mepitastatin and irinotecan into the cells, and the results showed that this model can be used as a glioma chip model for drug screening and release tests. Wang and coworkers developed a glioma-related microfluidic device with four parallel chambers to monitor rat C6 glioma cell responses to colchicine anticancer drugs. They observed significant changes in cell morphology and death rate upon increasing colchicine concentration or treatment time. This research will be helpful in developing glioma-related anticancer drugs and for developing glial cell-based biosensors for glioma detection^198^.

### Colorectal-tumor chip

Colorectal cancer (CRC) is the second leading cause of cancer-related death due to the high incidence of cancer metastasis and the low cure rate of chemotherapy. Endothelial cells lining in the microvasculature play a key gatekeeper role in avoiding colorectal cancer. Therefore, human colonic microvascular endothelial cells in the colon (HCoMECs) are often adopted to develop a microvascular system model^199^. Gemcitabine (2′,2′-difluoro-2′-deoxycytidine) (GEM) is often used in the treatment of advanced CRC^200,201^. GEM has a small molecular weight, high solubility in water, a short half-life in plasma, and a low concentration distribution around the tumor^201,202^. Mmp-1 functions as a collagenase in degrading ECM^203^, is thought to be related to the poor prognosis in advanced colon cancer and has become a target for tumorigenesis and the metastasis of various cancers^204^. Conversely, proangiogenic signals such as VEGF can promote processes such as proliferation, endothelial movement, and the expansion of filamentous pseudopodia. Preclinical and clinical data show that VEGF is a main angiogenic factor in human colon cancer and is associated with metastasis formation and adverse prognostic responses^205^. Various models have also been proposed to verify this relationship^206–208^.

The blood vascular system can mimic the human colorectal-tumor microenvironment, which reconstructs the physiological function of microvascular tissue^209^. To build such a system, Oliveira and Carvalho et al. proposed a 3D in vitro model and demonstrated it with a microfluidic chip. Microfluidic chips can create precise concentration gradients that are continuously modulated through the side channels of the surrounding vascular simulation system and therefore can assess the efficiency of drug delivery. It has been verified that nanomedicine can be effectively delivered in a gradient diffusion manner. In addition, the trend of the target gene can be used to analyze the gene expression level. Carvalho et al. developed a colorectal microfluidic chip with three main parts^210^. The central circular chamber is a hydrogel containing extracellular matrix (ECM) with embedded HCT-116 cells and has one inlet and one outlet. The other two perfused channels are located on either side of the circular central chamber. CRC

cells, labeled in red, and HCoMECs, in yellow, respond to the incoming VEGF growth factor. The microvascularized colorectal-tumor-on-a-chip model was developed to investigate sprouting formation and drug screening applications.

### Breast tumor chip

There is a large difference in morphology between 2D and 3D cultures of breast tumor cells. In 2D culture, nonmalignant cells are morphologically similar to malignant cells. However, in 3D culture, nonmalignant breast tissue cells of are polar and arranged in a tube, and the malignant cells form spherical tissue. The researchers compared the effects of several antitumor drugs, such as paclitaxel, olaparib, and cisplatin, in 2D- and 3D-cultured breast tumor cells and found that the drug response was weakened in 3D-cultured tumor cells. Therefore, researchers propose that 3D-cultured breast tumor chips can be used to predict antitumor drug responses^211,212^. In breast cancer, the frequency of bone metastases is ~70%. Therefore, studying bone metastases from breast tumors is also an important subject. There is a report of a 3D bone chip with mineralized collagen bone tissue growing naturally (Fig. 6a)^213^. Based on this chip, a bone metastasis model of breast tumor cells was established. Metastasis was evaluated by examining the tissues of cocultured breast tumor metastatic cells and osteoblasts. This model provides a new in vitro model for studying bone metastases from breast tumors^213^.

### Pancreatic tumor chip

Since pancreatic cancers are invasive solid tumors with hypovascular structures and extensive fibrosis^214^, developing pancreatic tumor-on-a-chip systems for in vitro experiments is essential for enhancing pancreatic drug screening efficiency before animal model testing and clinical trials. Compared with those in the monolayer model, the cells in the spheroid-based 3D model exhibit higher drug resistance during drug testing, and the 3D model is closer to the chemical environment of native tissues^215^. Nishiguchi et al. investigated a pancreatic cancer model with a blood capillary structure that is able to replicate cellular morphologies and reflect the death of endothelial cells during the metastasis process^216^. A layer-by-layer technique was proposed to construct the 3D tumor model in later development, especially to mimic the tumor microenvironment and study cancer metastasis progression. This in vitro 3D layer-by-layer model could be integrated with the blood vascular system to build blood capillary models, which are used for assessing the efficacies of different drugs against pancreatic cancer cells, such as the hematogenous metastases of MIA PaCa-2 pancreatic cancer cells and the lymphogenous metastases of BxPC3 pancreatic cancer cells. Zhao et al. studied the metastasis of pancreatic cancer by culturing two independent organs in a biomimetic vascular system (Fig. 6c)^217^. This vascularized MSC microenvironment provided a suitable platform for pancreatic cancer cell growth.

## Application of tumor modeling

### Modeling organ-level physiology and disease

An organ is a hierarchical structure composed of two or more different tissues. The key step in replicating the functions at the organ level is to combine two or more different tissue types. In addition, the tissue itself is composed of different cell types. Therefore, although the culture of a single cell type can mimic some aspects of the tissue microenvironment on a chip, it is usually not enough to produce organ-like functions. The development of tumor-on-a-chip technology has a great ability to replicate the tumor microenvironment in vitro. Improved tumor modeling has great potential for studying the basic mechanisms of organ physiology and disease.

**Fig. 7:**
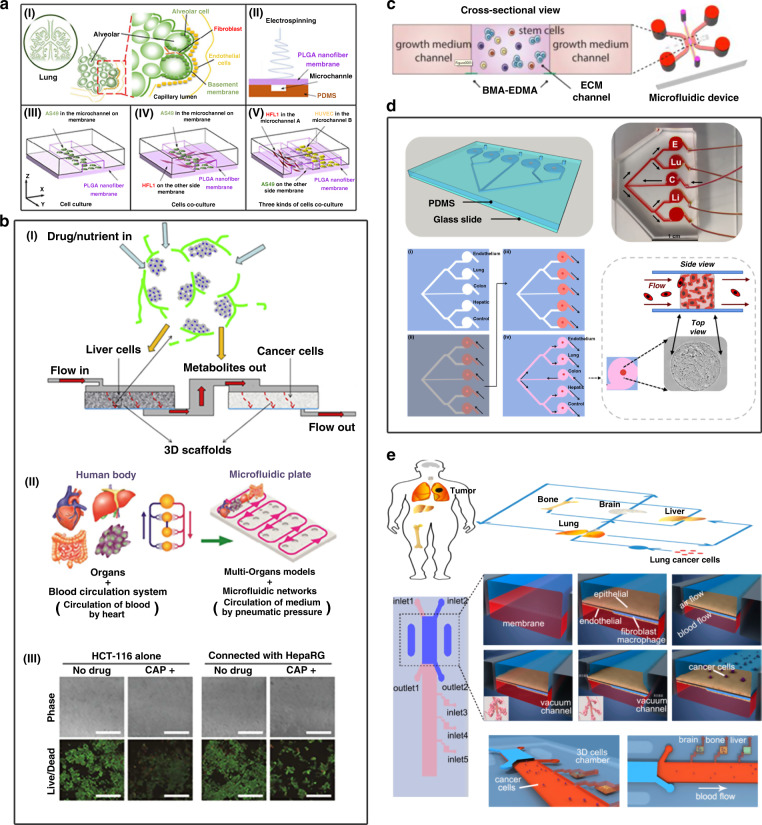
Applications of tumor-on-a-chip systems. **a** Based on the alveolar microenvironment, a lung organ chip was developed for lung cancer research. I Alveolar structure. II Schematic diagram of the preparation of the PLGA nanofiber membrane microfluidic chip by electrostatic spinning. III Schematic diagram of A549 cells cultured on chip. IV Schematic diagram of the coculture of A549 and HFL1 cells on a chip. V Schematic diagram of the coculture of A549, HFL1 and HUVECs on a chip. Adapted with permission^80^. Copyright 2018, The Royal Society of Chemistry. **b** Multiorgan system platforms for modeling tumor morphology and testing drug response. I 3D model device that aggregates liver cells and cancer cells for drug testing^219^. II Model of a multiorgan system on a plate for multithroughput. III HCT-116 cell activity was detected on the 3rd day of culture. Green: living cells; Red: dead cells. Scale bars: 500 µm^9^. Adapted with permission^219^ and ^9^. Copyright 2012, Biomaterials Publishing Group and Copyright 2018, The Royal Society of Chemistry, respectively. **c** Schematic of the cross-sectional view of the 3D stem cell differentiation assay using BMA-EDMA-based hydrogel patterning. Adapted with permission^223^. Copyright 2018, Wiley. **d** The design and biofabrication of a metastasis‐on‐a‐chip system for evaluating the metastatic preference of cancer cells. Adapted with permission^117^. Copyright 2019, American Chemical Society. **e** Design and preparation of a multiorgan microfluidic chip to mimic the in vivo microenvironment of lung cancer metastasis. The chip was made of PDMS, consisting of an upstream “lung organ” and three downstream “distant organs”. Different types of cells, including bronchial epithelial cells, stromal cells and cancer cells, were cocultured in the system, and some physical factors, such as air and pressure, were applied to the system to reconstitute the tissue-tissue interfaces and dynamic microenvironments. Adapted with permission^225^. Copyright 2016, American Chemical Society.

Many tumor-on-a-chip models have been developed. Lung tumor modeling is one of the hotspots in constructing organ chips. Earlier, Huh et al.^131^ used a bionic microsystem to reconstruct the alveolar–capillary interface of the key function of the human lung. This model can produce a complex, integrated organ-level response to bacteria and inflammatory cytokines entering the alveolar space. It has been demonstrated that organ microdevices on a chip with mechanical activity can reconstruct tissue–-tissue interfaces that are critical to organ function. Yang et al. used a polylactic acid-glycolic acid (PLGA) electrospun nanofiber membrane to prepare a low-carbon silicon wafer based on the alveolar microenvironment (Fig. 7a)^80^. The 3 μm PLGA nanofiber membrane has good controlled porosity, molecular permeability and biocompatibility and can be used to simulate the alveolar respiratory membrane. Lung tumor models are mainly cell cultures and cocultures of human non-small-cell lung cancer cells (A549), human fetal lung fibroblasts (HFL1), and targeted epidermal growth factor receptor (EGFR). In this model, the researchers explored possible sources of drug resistance in A549 cells in the presence of HFL1 cells. A549, HFL1, and human umbilical vein endothelial cells (HUVECs) were cultured together, and the results showed that A549 cells can cause endothelial cell apoptosis or death, which in turn causes tumor invasion. The lung cancer model is simple, effective and convenient to operate. It has a potential role in the personalized treatment of lung tumors, other clinical treatments and tissue engineering.

### Modeling tumor morphology and drug response with high fidelity

It is fundamental that tumor-on-a-chip systems recapitulate in vivo tumors to improve anticancer drug screening strategies. In 2014, Vidi et al. developed a breast-on-a-chip platform consisting of a breast luminal epithelium monolayer on a semicircular acrylic support to mimic cancer mammary ducts^218^. Compared with tumor cells cultured in the traditional planar monoculture method, tumor cells grown in channels have different morphologies and display different sensitivities to anticancer drugs. These findings provide new insights for the design and testing of cancer treatments. Ma et al. established a two-chamber (3D-mPTC) tissue model based on three-dimensional microscale perfusion in early research, which was combined with the cytotoxicity of liver metabolism anticancer drugs for detection, and realized the most basic organ chip model (Fig. 7b)^219^. Later, Satoh et al. established a two-organ system model composed of liver and tumor^9^. In this model, the proliferation of HCT-116 cancer cells was inhibited by capecitabine (CAP) and its metabolite 5-fluorouracil (5-fu). The effects of 5-fu and 5-fu precursors (CAP and tegafur) on multiple organ models (including cancer) and connective tissues were also investigated. Researchers have also produced microenvironment chips for breast cancer, normal liver tissue or tumor-bearing liver tissue, which simulate the microenvironment in the chip to study the dynamic and spatial transmission of particles. Compared with blood vessels in the healthy liver microenvironment, breast cancer cells cultured in the microenvironment of this model have higher vascular porosity and tumor microenvironment permeability. This model can be used to determine the microenvironment affected by tissues or tumors or the properties of specific drugs and nanoparticles, including transport, efficacy, and selectivity, and can achieve optimal treatment in a dynamic, high-throughput manner^9,220^.

### Modeling cancer invasion and metastasis

Tumor metastasis is one of the main challenges of current clinical cancer treatment. It can often cause high patient mortality and is responsible for more than 90% of all cancer-related deaths^221,222^. Currently, most tumor-on-a-chip systems mimic tumors only in situ; thus, the mechanism of tumor cell metastasis is still unclear, particularly the reasons for the initial activation of tumor cell growth and metastasis (such as specific signaling pathways) and the role of the microenvironment in regulating this phenomenon. Therefore, it is very important to use experimental models to effectively characterize the metastatic microenvironment (Fig. 7c, d)^117,223^. Skardal’s group demonstrated the utility of a 2-organoid metastasis-on-a-chip (MOC) platform^224^. By using microfluidics to provide circulating flow through the organoid system, tumor cells grow in the primary focus and enter the circulation, with the result that CRC cells of the colon organoids diffuse into the circulation, and then the transferred cells settle on the downstream liver organoids. This model was one of the first in vitro models to simulate tumor cell metastasis, reproducing metastasis from a three-dimensional primary tissue to a three-dimensional target tissue. Recently, the same group enriched the tumor metastasis model by adding additional functionality, such as expanding the downstream organoids from one site to four sites. They developed a multisite metastasis-on-a-chip platform to assess the metastatic preference of cancer cells^117^. Researchers have used 3D photopatterning technology to fabricate chips with multiple 3D organoids. Specifically, cancer cells start from colorectal cancer (CRC) organoids located in a single microfluidic chamber connected to multiple downstream cavities in which liver, lung, and endothelial structures are housed. In this system, under the circulating flow of liquid, it is found through fluorescence imaging tracking that HCT-116 CRC cells preferentially enter the liver and lung structures, which are the corresponding organs with the most CRC metastasis in human patients. The platform can help to better understand the mechanism of metastasis and has the potential to lead to the identification of targets for intervention. Wang’s group took advantage of another multiorgan-on-a-chip platform to study lung cancer metastasis (Fig. 7e)^225^. The system consists of four organs: one upstream lung and three downstream parallel brain, bone, and liver organs. This model simulated the metastasis of lung cancer to the brain, bone, and liver.

Migration and invasion research studies in tumor-on-a-chip models have been improved on the basis of more traditional analysis methods (such as Transwell culture and wound scratching)^104,112^. Toh et al. described a microfluidic tumor-on-a-chip cell migration model incorporating a microfluidic system to understand the progress of cell intravasation^226^. This platform integrates a 3D microenvironment that plays a key role in the invasiveness of cancer cells, creating an attractive model for antimigration and anti-invasive cancer drug testing that can be multiplexed for high-throughput analysis. There is a great need to develop new tumor models to improve cancer management and the prognosis of cancer patients. As a result, such models may ultimately help reduce healthcare costs.

### Tumor models for drug screening

**Fig. 8:**
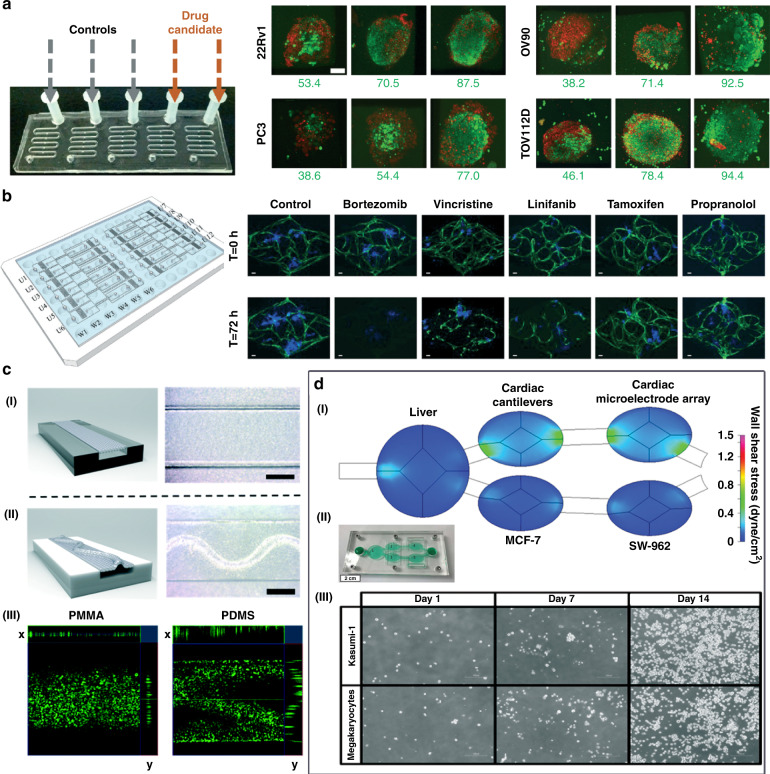
Illustration of tumor-on-a-chip devices applied to drug screening. **a** The microdissected tumor/tissue samples (MDTs) were incubated in independent chambers of a microfluidics chip to assess one or several selected drug candidates. Some were treated with drugs, and the others were kept as nontreated controls. MDTs were labeled with viability dyes: CTG (live, green) and PI (dead, red). Adapted with permission^121^. Copyright 2016, The Royal Society of Chemistry. **b** A vascularized and perfused organ-on-a-chip platform for large-scale drug screening has been applied to some FDA-approved anticancer drug-blinded screenings, successfully identifying both antiangiogenesis and antitumor drugs. Adapted with permission^86^. Copyright 2017, The Royal Society of Chemistry. **c** Comparison diagram of the microfluidic chip models made by PMMA and PDMS. I The PMMA-PETE device with flat PETE membrane. II The PDMS-PETE device with wrinkled PETE membrane. III Confocal microscopic images of human lung adenocarcinoma cells cultured on PETE membrane in the PMMA and PDMS devices and stained with calcein. Adapted with permission^164^. Copyright 2019, The Royal Society of Chemistry. **d** The five-chamber reconfigurable multiorgan system platform used to evaluate the efficacy and off-target toxicity of anticancer drugs. I Computational fluid dynamics modeling. II Photograph of the multi-organ system. III Representative phase contrast images of Kasumi-1 and megakaryocytes growing inside the multi-organ system chamber on days in vitro (DIV) 1, 7, and 14. Adapted with permission^230^.

Complex 3D organs are not well represented by monolayer cell models, the standard format for many drug screens. Therefore, organ technology is widely used in tumor drug detection. In recent years, researchers have designed a variety of tumor-on-a-chip models for drug testing (Fig. 8)^86,121,227^. The model is continuously optimized to identify drugs with good efficacy and low toxicity. The microphysiological system (MPS), for example, is being developed with the goal of being able to capture the complexity of in vivo physiology. Gervais et al. made use of a simple microfluidic platform that can reliably trap samples to incubate eight different types of microdissected tissues (MDTs) in a low-shear stress environment. Through this platform, the researchers analyzed MDT viability by confocal microscopy and flow cytometry, providing information on chemosensitivity testing and drug response (Fig. 8a)^121^. Phan et al. created arrayed vascularized microtumors (VMTs) and used them for blind-hole screening (Fig. 8b)^86^. They can analyze small compound libraries, including FDA-approved compounds, and successfully identify antitumor drugs. This 3D platform is suitable for the efficacy/toxicity screening of various tissues under more complex conditions than physiological environments.

Compared with the traditional polydimethylsiloxane (PDMS) chip model, the organ chip using PMMA has more reliable cytotoxicity results, which has important research applications in drug screening. Nguyen et al. used (3-glycidyloxypropyl) trimethoxysilane to bond polymethyl methacrylate (PMMA) polyethylene terephthalate (PETE) orbital etching film (GLYMO), which can be used in microfluidic devices that are not permeable to small molecules and can enable reliable cytotoxicity tests, such as those for anticancer drugs (Fig. 8c)^164^. The bonding strength between the two substrates is sufficient for culture exchange, and even at a gauge pressure higher than 135 kPa, the fluid can still pass through the device without leakage. PMMA organ chips have been successfully demonstrated to show more reliable cytotoxicity results for vincristine using human lung adenocarcinoma cells. This method uses a variety of thermoplastics and porous orbital etching to expand simple membrane manufacturing capabilities, create three-dimensional microstructure physiological conditions, and more accurately simulate organ levels.

The multiorgan chip system can be used not only for drug screening but also for predicting preclinical target efficacy, metabolic conversion rate, and target off-target toxicity^228^. Hickman et al. took advantage of a pumpless 4-organ system (liver, cardiac, neuronal, and muscle) to evaluate the human response to five different drugs for 14 days. The system worked under continuous flow conditions in a serum-free defined medium. This technology provided a novel tool to improve the predictive ability of preclinical efficacy/toxicity studies^229^. Subsequently, Hickman’s group found that antileukemia drugs can be tested by coculturing primary human hepatocytes and human bone marrow cell cultures of two types of cancer in a chip model (Fig. 8d)^230^. Diclofenac and imatinib have cytostatic effects on bone marrow. Anti-imatinib does not affect liver vitality, and diclofenac reduces liver vitality by 30%. Multiple drugs have been evaluated in organ models of multidrug-resistant vulvar cancer lines with non-multidrug-resistant breast cancer cells, primary liver cells, and cardiomyocytes from induced pluripotent stem cells. Tamoxifen reduces the activity of breast cancer cells only after producing metabolites but does not affect vulvar cancer cells. The combined use of tamoxifen and verapamil will produce nontargeted cardiac effects, which are manifested in reduced contractility, decreased beating frequency, and slower conduction speed but do not affect viability. These models show that cell-based in vitro culture systems can be used to evaluate the target efficacy and target toxicity of the parent drug and its metabolites and can improve the efficiency of drug evaluation in preclinical studies. By building a better predictive model, organ chip model technology significantly reduces research and development costs between preclinical and human trials. This technology is determined by changes in the direct cost, success rate, and duration of the research and development process and may become a challenge for future healthcare systems, including the provision of innovative treatments and expensive drugs^231^.

## Conclusions and future perspectives

In this review, we have discussed the currently available tumor-on-a-chip platforms and their potential applications for cancer biology and treatment. Compared with conventional in vitro planar cell models and in vivo animal models, tumor-on-a-chip systems can mimic the complexity of in vivo tumor masses, which are considered to be reliable for the development of effective anticancer therapies. To provide high-throughput and modular tumor-on-a-chip systems, microfluidic technologies are also introduced, aiming at reconstructing in vivo-like environments in a more reliable way for cancer research. Tumor-on-a-chip platforms can mimic the main in vivo TME features, exhibiting great promise as more realistic and accurate models for studying the metastasis, distribution and mechanisms of tumor growth, as well as drug toxicity and therapeutic efficacy. The superiority of tumor-on-a-chip platforms as candidates for conventional preclinical models has attracted worldwide research attention, and great amounts of scientific progress have been made. A large number of tumor-on-a-chip platforms have been designed and established, simulating tumors in the lung, liver, breast, and brain. They are mainly applied to screen anticancer drugs and perform fundamental research on cancer metastasis to understand the biological basis of cancer. As an alternative to conventional preclinical models, tumor-on-a-chip platforms have the potential to improve many fields of basic research and drug development. This review examines tumor-on-a-chip microfabrication technology, classifications and applications in greater detail than previous reviews, helping promote the broader adoption of this platform.

However, due to the complexity of the physiological structure and microenvironment in vivo, tumor chips still face many challenges before widespread integration of these platforms into practical pharmaceutical industrial and clinical applications: (i) Simulation of complex signal regulation functions. Although tumor-on-a-chip platforms can mimic the in vivo TME and even establish a body-on-a-chip system through connections with organ-on-a-chip devices, it is difficult to simulate the various complex signal regulation responses of other nonadjacent organs in the human body to cancer, especially signals from the endocrine system or the immune system. (ii) The industrial manufacture of microfluidic devices and their data standardization. Tumor-on-a-chip systems are based on esoteric microfabrication technologies, and there is a great need to explore how to rationally design microfluidic devices for precise control of the physicochemical properties of the chips. Moreover, not all researchers or users are proficient in microfabrication facilities and the related expertise; therefore, it is important to develop user-friendly on-chip systems and simultaneously standardize the data from different laboratories so that researchers who are not experts can immediately use these emerging models for research and obtain meaningful data for clinical translation. (iii) Exploration of new biocompatible materials. Polydimethylsiloxane (PDMS) is the most widely used material to fabricate tumor-on-a-chip devices; however, PDMS is known to easily adsorb hydrophobic compounds, such as drugs and proteins, which may reduce effective drug concentrations and activity and can cause experimental errors, limiting its application. A variety of candidate materials have been developed, but these devices need to be optically transparent, easily moldable, mechanically adjustable, nonreactive and economical, limiting the available candidates. In conclusion, although tumor-on-a-chip platforms still face many challenges, they are promising platforms to facilitate the development of cancer therapies. To achieve these goals, interdisciplinary cooperation is needed among researchers from material science, biomedical engineering, cell biology, biophysics and oncology to make concerted efforts in designing and optimizing tumor-on-a-chip systems for cancer research and drug discovery, finally translating bioinspired designs to clinical applications.
